# Animal models mimicking aminoglycoside-induced renal damage

**Published:** 2016-01-06

**Authors:** Sandra Rodríguez Salgueiro, Lucía González Núñez

**Affiliations:** ^1^Electron Microscopy Laboratory, National Center for Scientific Research, Havana, Cuba; ^2^Human Morphophysiology Department, Latinamerican School of Medicine, Havana, Cuba

**Keywords:** Animal models, Nephrotoxicity, Aminoglycosides, Kanamycin

**Implication for health policy/practice/research/medical education:**

Models of nephrotoxicity in rats and mice are essential to achieve a better understanding of the pathophysiology of aminoglycoside-induced damage and to consider strategies for the protection, repair and regeneration, which could be further extrapolated for their use in the clinic.

Aminoglycoside antibiotics consisting gentamicin, kanamycin, amikacin and other can cause acute kidney injury (1). This is a common problem in intensive care medicine (2). It could be fatal (3) or can progress towards chronic kidney disease in survivors (4).

Knowledge of the possible mechanisms involved in aminoglycoside-induced nephrotoxicity is largely due to the use of experimental animals (5). Animal models based on treatments with aminoglycosides have also been used to evaluate possible protective or regenerative strategies (6,7).

The development of aminoglycoside-induced toxicity models is a complex task, because these compounds can provoke animal death due to renal toxicity and therefore all the adverse effects associated with chronic administration of the aminoglycoside cannot be ascertained (8).

Rats are usually used in studies of aminoglycoside-induced nephrotoxicity, due to their similarity to human beings in terms of the histological structure of the kidneys (9). The deleterious effect of aminoglycosides on rat renal cortex is currently well characterized (5). Although renal histological structure of mice is similar to the rats, they have been used to a lesser extent (10,11).

Nephrotoxicity models commonly use treatments with high doses of an aminoglycoside over a short period of time to induce acute renal injury (12). The acute models have allowed knowing the changes that aminoglycosides induce in the kidney. Epithelial cells of the proximal tubules are the major site of accumulation of these compounds (13).

Although non metabolized aminoglycosides are excreted unchanged through the kidneys, a fraction of filtered aminoglycoside is reabsorbed by proximal tubular cells and causes its toxic effect (5). Nephrotoxicity due to aminoglycosides has been largely recognized as a tubule-interstitial damage, without significant changes in glomeruli. It should be attributed to an early evaluation of the kidneys, as these studies are performed mainly through acute models, in which glomerular effects are not still evident (14).

More recently, it has been shown that aminoglycosides can also induce glomerular lesions, such as congestion, glomerular hypertrophy or atrophy (6), accumulation of amorphous substance in mesangial matrix (15), decreased glomerular filtration rate and other intra-glomerular alterations (5,16).

Since some patients surviving after acute kidney injury can progress towards chronic kidney disease (4), chronic models of aminoglycoside induced toxicity have also been generated. In these models, the evaluation of the state of the kidney is performed later, during the recovery phase following the acute renal damage (11,17).

In chronic models the tubular damage prevails over glomerular damage. These models mimic the clinical condition in which patients are in the recovery phase of renal morphophysiology after acute kidney injury caused by aminoglycosides (13) and at the same time, allow us to study what happens in the recovery phase. This could be the most appropriate phase to intervene in survivors of acute kidney injury, since progression towards chronic kidney disease has been observed even several months after the initial injury (18).

Gentamicin is the aminoglycoside of choice in most experimental models (19). However, with the use of kanamycin, morphological changes in glomeruli, proximal tubules and interstitium have been described similar to that obtained in studies with gentamicin (11,17). In addition, other glomerular effects of these antibiotics have been described through the use of chronic models of kanamycin nephrotoxicity in rats, in terms of increased mesangial matrix at the expense of increasing the number of mesangial cells and also the presence of glomerular synechiae (17). The presence of synechiae is consistent with the sub-capsular accumulation of filtered proteins, leading to the adhesion of the denuded glomerular basement membrane to the Bowman’s capsule (20). Both glomerular synechiae and mesangial expansion lead to capillary obstruction and this affects the intensity of glomerular filtration (21).

The morphological changes in the renal cortex after acute and chronic models of aminoglycoside nephrotoxicity have been described as patchy, due to the simultaneous occurrence of necrosis and regeneration (22,23).

Acute models of gentamicin nephrotoxicity have been used to evaluate many protecting candidates (6,24). Chronic models based on treatments with kanamycin have also been used to evaluate possible protective agents addressed to accelerate the recovery of renal morphophysiology after acute renal injury (11,17).

## Conclusion

In conclusion, models of nephrotoxicity in rats and mice are essential to achieve a better understanding of the pathophysiology of aminoglycoside-induced damage and to consider strategies for the protection, repair and regeneration, which could be further extrapolated for their use in the clinic (25,26).

## Authors’ contribution

All authors wrote the paper equally.

## Conflicts of interest

The authors declared no competing interests.

## Ethical considerations

Ethical issues (including plagiarism, data fabrication, double publication) have been completely observed by authors.

## Funding/Support

None.
